# Intratumoral pan-ErbB targeted CAR-T for head and neck squamous cell carcinoma: interim analysis of the T4 immunotherapy study

**DOI:** 10.1136/jitc-2023-007162

**Published:** 2023-06-15

**Authors:** Sophie Papa, Antonella Adami, Michael Metoudi, Richard Beatson, Molly Sarah George, Daniela Achkova, Evangelia Williams, Sefina Arif, Fiona Reid, Maria Elstad, Nicholas Beckley-Hoelscher, Abdel Douri, Marc Delord, Mike Lyne, Dharshene Shivapatham, Christopher Fisher, Andrew Hope, Sakina Gooljar, Arindam Mitra, Linda Gomm, Cienne Morton, Rhonda Henley-Smith, Selvam Thavaraj, Alice Santambrogio, Cynthia Andoniadou, Sarah Allen, Victoria Gibson, Gary J R Cook, Ana C Parente-Pereira, David M Davies, Farzin Farzaneh, Anna Schurich, Teresa Guerrero-Urbano, Jean-Pierre Jeannon, James Spicer, John Maher

**Affiliations:** 1 School of Cancer & Pharmaceutical Sciences, King's College London, London, UK; 2 Department of Medical Oncology, Guy's and St Thomas' NHS Foundation Trust, London, UK; 3 Department of Infectious Diseases, School of Immunology & Microbial Sciences, King's College London, London, UK; 4 Department of Immunobiology, School of Immunology & Microbial Sciences, King's College London, London, UK; 5 School of Life Course & Population Sciences, King's College London, London, UK; 6 Department of Biostatistics and Health Informatics, Institute of Psychiatry Psychology & Neuroscience, King's College London, London, UK; 7 Guy’s and St Thomas’ Biomedical Research Centre, Guy's and St Thomas' NHS Foundation Trust and King’s College London, London, UK; 8 Good Manufacturing Practice Unit, Guy’s and St Thomas’ Biomedical Research Centre, Guy's and St Thomas' NHS Foundation Trust, London, UK; 9 Head and Neck Pathology, Guy's and St Thomas' NHS Foundation Trust, London, UK; 10 Faculty of Dentistry, Oral and Craniofacial Sciences, Guy's Hospital, King's College London, London, UK; 11 Department of Nuclear Medicine, Guy's and St Thomas' NHS Foundation Trust, London, UK; 12 London School of Biomedical Engineering and Imaging Sciences, King's College London, London, UK; 13 Department of Head and Neck Oncology, Guy's and St Thomas' NHS Foundation Trust, London, UK; 14 Department of Immunology, Eastbourne Hospital, Eastbourne, UK; 15 Leucid Bio Ltd, London, London, UK

**Keywords:** Cell Engineering, Clinical Trials as Topic, Head and Neck Neoplasms, Immunotherapy, Adoptive, Therapies, Investigational

## Abstract

**Background:**

Locally advanced/recurrent head and neck squamous cell carcinoma (HNSCC) is associated with significant morbidity and mortality. To target upregulated ErbB dimer expression in this cancer, we developed an autologous CD28-based chimeric antigen receptor T-cell (CAR-T) approach named T4 immunotherapy. Patient-derived T-cells are engineered by retroviral transduction to coexpress a panErbB-specific CAR called T1E28ζ and an IL-4-responsive chimeric cytokine receptor, 4αβ, which allows IL-4-mediated enrichment of transduced cells during manufacture. These cells elicit preclinical antitumor activity against HNSCC and other carcinomas. In this trial, we used intratumoral delivery to mitigate significant clinical risk of on-target off-tumor toxicity owing to low-level ErbB expression in healthy tissues.

**Methods:**

We undertook a phase 1 dose-escalation 3+3 trial of intratumoral T4 immunotherapy in HNSCC (NCT01818323). CAR T-cell batches were manufactured from 40 to 130 mL of whole blood using a 2-week semiclosed process. A single CAR T-cell treatment, formulated as a fresh product in 1–4 mL of medium, was injected into one or more target lesions. Dose of CAR T-cells was escalated in 5 cohorts from 1×10^7^−1×10^9^ T4^+^ T-cells, administered without prior lymphodepletion.

**Results:**

Despite baseline lymphopenia in most enrolled subjects, the target cell dose was successfully manufactured in all cases, yielding up to 7.5 billion T-cells (67.5±11.8% transduced), without any batch failures. Treatment-related adverse events were all grade 2 or less, with no dose-limiting toxicities (Common Terminology Criteria for Adverse Events V.4.0). Frequent treatment-related adverse events were tumor swelling, pain, pyrexias, chills, and fatigue. There was no evidence of leakage of T4^+^ T-cells into the circulation following intratumoral delivery, and injection of radiolabeled cells demonstrated intratumoral persistence. Despite rapid progression at trial entry, stabilization of disease (Response Evaluation Criteria in Solid Tumors V.1.1) was observed in 9 of 15 subjects (60%) at 6 weeks post-CAR T-cell administration. Subsequent treatment with pembrolizumab and T-VEC oncolytic virus achieved a rapid complete clinical response in one subject, which was durable for over 3 years. Median overall survival was greater than for historical controls. Disease stabilization was associated with the administration of an immunophenotypically fitter, less exhausted, T4 CAR T-cell product.

**Conclusions:**

These data demonstrate the safe intratumoral administration of T4 immunotherapy in advanced HNSCC.

**WHAT IS ALREADY KNOWN ON THIS TOPIC**

- ErbB-targeted therapies such as cetuximab and afatinib have antitumor activity in head and neck squamous cell carcinoma (HNSCC).

**WHAT THIS STUDY ADDS**

- This study demonstrates the safety of intratumoral administration of panErbB-targeted chimeric antigen receptor T-cells (CAR T-cells) in patients with locally advanced or recurrent HNSCC. Disease stabilization was achieved in the majority of treated patients, suggestive of an efficacy signal.

**HOW THIS STUDY MIGHT AFFECT RESEARCH, PRACTICE OR POLICY**

- Following intratumoral delivery, lack of leakage of potentially toxic CAR T-cells into the circulation provides a rationale for further potentiation of this approach, for example, through CAR T-cell armoring strategies.

## Introduction

Head and neck squamous cell carcinoma (HNSCC) most commonly presents with locally advanced disease.^1^ Despite multimodality treatment, locoregional failure is frequent and 5-year survival falls below 50% for those who present with locally advanced tumors.^1^ Once patients become unsuitable for conventional treatment options, prognosis is dismal with 100% mortality within 30 weeks.^2^

Immunotherapy using chimeric antigen receptor-engineered T-cells (CAR-T) has achieved unprecedented success against selected hematological cancers, most notably B-cell malignancy.^3^ However, solid tumors such as HNSCC are much more resistant to this approach.^4^ Epidermal growth factor (EGF) receptor is expressed in 90% of tumors while other members of the ErbB family are commonly also present.^5^ To exploit this, we developed a CAR termed T1E28z in which the promiscuous ErbB ligand, T1E,^6^ (a chimera derived from transforming growth factor α and EGF) is fused to a CD28 spacer, transmembrane and endodomain followed by a CD3ζ endodomain.^7^ Conscious of the challenge to achieve efficient retroviral transduction of T-cells from patients with advanced cancer, we coexpressed T1E28z with the 4αβ chimeric cytokine receptor. 4αβ allows interleukin (IL)-4-driven selective expansion of transduced cells during manufacture.^8^ When T1E28z and 4αβ are coexpressed in patient T-cells, the resultant drug product is referred to as T4 immunotherapy. Preclinical evaluation demonstrated that both T1E28z- and T4-engineered CAR-T proved efficacious in models of several solid tumor types, including HNSCC.^7 9–11^ Moreover, intratumoral CAR T-cell delivery has proven to be safe in mouse models, owing to retention of CAR T-cells within the tumor.^9 12^ Consequently, we undertook a first in human clinical trial of T4 immunotherapy in patients with HNSCC, employing intratumoral delivery to derisk this approach in man. This report describes an interim analysis of the dose escalation phase of the study.

## Methods

### Study design

This is an open-label, investigator-initiated, single-center (Guy’s and St Thomas’ NHS Foundation Trust, (GSTT)) non-randomized phase 1 study (EudraCT Number: 2012-001654-25). A traditional 3+3 dose escalation design was adopted without lymphodepleting chemotherapy. All 17 enrolled subjects provided written informed consent to participate. Subjects were not screened for tumor ErbB status prior to trial entry.

This report describes an interim analysis of the dose escalation portion of the trial. Primary objectives were to define dose-limiting toxicities (DLTs) of T4 immunotherapy in HNSCC and to determine a safe and feasible recommended dose for phase-2 testing. Secondary objectives were to investigate serum cytokine levels post T4 immunotherapy; assess persistence of CAR T-cells at the site of injection and efflux into the circulation; to undertake a preliminary assessment of efficacy and to determine effects on tumor ErbB (EGF receptor) expression.

### Subjects

Eligible subjects were >18 years of age with locally advanced and/or recurrent HNSCC, with or without metastatic disease (excluding brain metastases) for whom no standard therapy remained or was suitable. Additional inclusion criteria included disease measurable by response evaluation criteria in solid tumors (RECIST) V.1.1^13^; Eastern Cooperative Oncology Group (ECOG) performance status of 0–2; normal cardiac function; adequate bone marrow function (neutrophils >1.5×10^9^ /L, platelets >100×10^9^ /L, hemoglobin >90 g/L); international normalized ratio <1.5; serum creatinine <1.5 upper limit of normal (ULN); bilirubin <1.25 times ULN and alanine aminotransferase and aspartate aminotransferase <2.5 times ULN (<5 times ULN if liver metastases present).

Exclusion criteria included the presence/imminent occurrence of airway obstruction, unless tracheostomy in place; the presence/imminent occurrence of tumor-mediated infiltration of major blood vessels; infection with HIV-1, HIV-2, human T-lymphotropic virus (HTLV)-1, HTLV-2, hepatitis B, hepatitis C or syphilis; splenectomy; clinically active autoimmune disease; concurrent anticoagulation therapy or treatment in the week prior to T4 immunotherapy with: (1) systemic corticosteroids (>20 mg prednisolone/day); (2) any systemic immunomodulatory agent; (3) radiotherapy; (4) chemotherapy or (5) any investigational medicinal product.

### Manufacture and release of T4 immunotherapy

T4 immunotherapy was manufactured over 14 days using whole blood (40–120 mL) as starting material. Manufacture was undertaken at the GSTT GMP manufacturing facility as described,^14^ using immobilized CD3 and CD28 antibodies rather than paramagnetic beads to elicit T-cell activation. In brief, gene transfer was achieved using the SFG T4 retroviral vector, produced by EUFETS (now BioNTech; Iadr-Oberstein, Germany) from a PG13 master cell bank.^7^ Vector was harvested in serum-free X-VIVO 10 medium (Lonza, Basel, Switzerland), with batch titers from 2.65 to 2.9×10^6^ viral particles/mL. A vector multiplicity of infection of 4.2 was used to achieve gene transfer into activated T-cells. Interleukin-4 was the sole cytokine support provided after gene transfer.^14^ The drug substance was subject to release testing as summarized in online supplemental table S1.^15^ Drug product was suspended in X-VIVO medium (Lonza, Basel Switzerland) + 1X GlutaMAX (ThermoFisher Scientific, Horsham, UK) + 10% human AB serum (BioIVT, West Sussex, UK).

10.1136/jitc-2023-007162.supp1 Supplementary data

### Protocol treatment

Lidocaine or bupivacaine local anesthesia was administered as premedication to the overlying skin and target lesion(s). T4 immunotherapy was administered as a fresh product without prior lymphodepleting chemotherapy, meaning that vein to treatment time was 14 days. CAR T-cell doses ranged from 1×10^7^ to 1×10^9^ in five dose cohorts (online supplemental table S2).

Drug product was delivered in a bag containing a Luer port. The bag was carefully mixed to resuspend aggregates before the appropriate therapeutic volume (1–4 mL: online supplemental table S2) was drawn up into a Luer syringe.^14^ Following attachment of a 21 gauge needle, T4 immunotherapy was immediately delivered by intratumoral (single or multisite) injection to one or more predefined HNSCC tumor(s).^15^ In one case, ultrasound guidance was used to facilitate accuracy of injection.

### Endpoints

The primary endpoint was occurrence of DLTs induced by T4 immunotherapy within 6 weeks of treatment. Secondary endpoints included serum cytokine levels, presence of T4^+^ cells in the circulation and tumor biopsies (where available post therapy), effect of T4 immunotherapy on reactivity to MAGE-A3 and MAGE-A4 tumor antigens, objective tumor response and time to progression. Trafficking of T4 immunotherapy was assessed in a single subject, using a radiotracer consisting of [^111^In] Indium passively labeled T4^+^ T-cells (30×10^6^ cells containing 5MBq [^111^In] Indium). The radiotracer was administered on the same day as the T4 drug product, but as a separate procedure. A single photon emission CT (SPECT) scan was performed approximately 1 hour after radiotracer delivery while additional SPECT scans were performed at 24 and 48 hours and were coregistered with the initial CT scan.

### Response criteria

Response was assessed using RECIST V.1.1.^13^ Up to five accessible and measurable tumors were documented at baseline prior to injection with T4 immunotherapy. Baseline CT imaging was carried out a maximum of 4 weeks prior to T4 immunotherapy injection and response was evaluated by CT scanning 6 weeks after administration of T4 immunotherapy. Confirmation of any partial or complete response was required at least 4 weeks after initial response imaging. Ongoing evaluation after progression was by the referring clinician’s standard practice.

### Safety

Safety reporting was conducted using the National Cancer Institute Common Terminology Criteria for Adverse Events (NCI CTCAE V.4.03; https://evs.nci.nih.gov/ftp1/CTCAE/CTCAE_4.03/CTCAE_4.03_2010-06-14_QuickReference_8.5x11.pdf, accessed March 18, 2023). Decision to dose escalate was made after review of the preceding dose level safety outcomes by the combined trial steering and data monitoring committee.

### Statistical methods

All subjects who consented to treatment are described. All subjects who received T4 immunotherapy completed the minimum 28-day DLT analysis period and were included in the safety analysis. Descriptive statistics are presented to address the primary and secondary study aims. In exploratory comparative analyses, between-group comparisons were conducted by unpaired t-test, and within-group comparisons by paired t-test. Survival was analyzed using the Kaplan-Meier method. All statistical analyses were performed using GraphPad Prism V.9.5 (GraphPad software, Boston, Massachusetts, USA).

### Translational methods

Please see online supplemental methods section for additional translational methods.

#### Multiplex cytokine bead array

Serum levels of a custom panel of 29 cytokines were measured before, and at 30 min and 1, 4, 24, 48–96 and 120–168 hours post T4 immunotherapy. Frozen sera were thawed, vortexed, centrifuged at 1000×g for 10 min and analyzed undiluted using a ProcartaPlex Immunoassay Kit (ThermoFisher), according to manufacturer’s instructions. Data were analyzed using a Luminex xMAP Intelliflex (ThermoFisher).

#### Flow cytometric assays

All analyses were performed on a Fortessa flow cytometer (BD Biosciences, Franklin Lakes, New Jersey, USA) using FACSDIVA or FlowJo v10 software (both BD Biosciences).

##### Immunophenotyping of T4 immunotherapy product

Immunophenotyping was performed for information only on nine batches of T4 immunotherapy. Compensation beads were prepared fresh for every run and used as per manufacturer’s instructions, including fluorescence minus one controls. All incubations were performed on ice in the dark.

Drug substance (2 mL) was collected, centrifuged (200 g, 5 min) and resuspended at 10×10^6^/mL in PBS. For antibody panel 1, 100 µL of cells (1×10^6^) were stained with 3 µL biotinylated anti-hEGF antibody (R&D systems, code BAF236, Minneapolis, MN). Phosphate buffered saline (PBS; 2 mL) was then added, and the cells were washed (200 g for 3 min). After discarding supernatant, cells were resuspended in 100 µL PBS and incubated with 1 µL Streptavidin-PE (ThermoFisher, code S866) for 15 min. Wash was repeated and cells were resuspended in 100 µL PBS with 3 µL of each antibody (all from BioLegend, San Diego, CA; antiCD3 APC-Cy7 (HIT3a), antiCD4 AF700 (RPA-T4), antiCD8 FITC (SK1) antiCD62L PerCPCy5.5 (DREG-56), antiCCR7 BV605 (GOG3H7), antiCD57 APC (QA17A04), antiCD28 BV421 (CD28.2), antiCD45RO PE-Cy7 (UCHL1)) before being incubated for 25 min. Cells were washed and resuspended in 300 µL PBS for analysis. For panel 2, cells were similarly stained with anti-hEGF and Streptavidin-PE followed by 3 µL of each antibody (all from BioLegend; antiCD3 APC-Cy7 (HIT3a), antiCD4 AF700 (RPA-T4), antiCD16 FITC (3G8), antiCD25 PE (BC96), antiCD45RO PerCPCy5.5 (UCHL1), antiCD45RA BV605 (HI100), antiPD-1 APC (EH12.2H7), antiCD19 BV421 (HIB19), antiNKG2D PE-Cy7 (1D11)), incubated for 25 min, washed and resuspended in 300 µL PBS. For the unstained control, 50 µL of cells (5×10^5^) were taken washed once in PBS as per the above conditions, before being resuspended in 300 µL PBS for analysis.

Additional immunophenotyping was performed on a subset of T4 batches where sufficient retention samples were available. In these subjects, best RECIST response following T4 immunotherapy was stable disease (n=8) or progressive disease (n=3). Cryopreserved cells were thawed rapidly at 37°C, then washed with 1×PBS. Cells were surface stained with the following antibodies in 1×PBS for 30 min at 4°C (protected from light): CD4-APC-Cy7 (RPA-T4), CD8-Alexa Fluor 700 (SK1), PD-1-PerCP-CY5.5 (EH12.2H7), CD71-Per-CP-Cy7 (CY1G4), CD39-FITC (A1) (all BioLegend) and CD98-PE-Vio770 (REA387) (Miltenyi Biotec). CAR was stained with a biotinylated anti-hEGF antibody (R&D systems, code BAF236) followed by streptavidin-BV510. Cells were stained concurrently with 1 µL/mL Live/Dead dye (ThermoFisher). Cells were washed with 1×PBS, then fixed with FOXP3 fix buffer (ThermoFisher) for 20 min at room temperature (protected from light). Cells were stained for intracellular markers using the following antibodies in FOXP3 perm buffer: Glut1-APC (Abcam, Cambridge, UK), PGC1α-rabbit (Novus Biologicals, Bio-Techne) followed by APC-rabbit-IgG (BioLegend). Donor cells from a single healthy donor were analyzed in each run as a control for consistency.

##### CAR-T cell effector molecule production assay

Functional analysis was also performed on T4 retention samples described in the previous paragraph. Thawed CAR T-cells were resuspended at 1 million/mL in RPMI 1640 medium (ThermoFisher) supplemented with 10 mM glucose, 0.1 mM non-essential amino acids, 10 mM HEPES buffer, 1 mM sodium-pyruvate and 10% fetal calf serum (FCS; all Sigma-Aldrich). Brefeldin A (1 µg/mL; BioLegend) was added to block export of effector proteins. Cells were either rested or activated with plate bound CD3 (OKT3 1 µg/mL) and CD28 (CD28.2, 0.5 µg/mL) antibodies (all BioLegend) in a 96 well plate in an incubator overnight at 37°C, 5% CO_2._ After washing in 1×PBS, cells were surface stained as before with: biotinylated anti-hEGF antibody (R&D systems, code BAF236), Streptavidin-BV510, CD4-APC-Cy7, CD8-Alexa Fluor 700, and live/dead dye. After fixation with Cytofix (BD Biosciences) for 20 min at 4°C, cells were stained with Granzyme-B-AF488 (GRZB; BioLegend), tumor necrosis factor (TNF)-α-APC and IFN-γ-BV421 (all BD Biosciences) in permeabilization buffer (1% FCS, 0.1% saponin in 1×PBS) for 30 min.

## Results

### Trial participants

**Figure 1:**
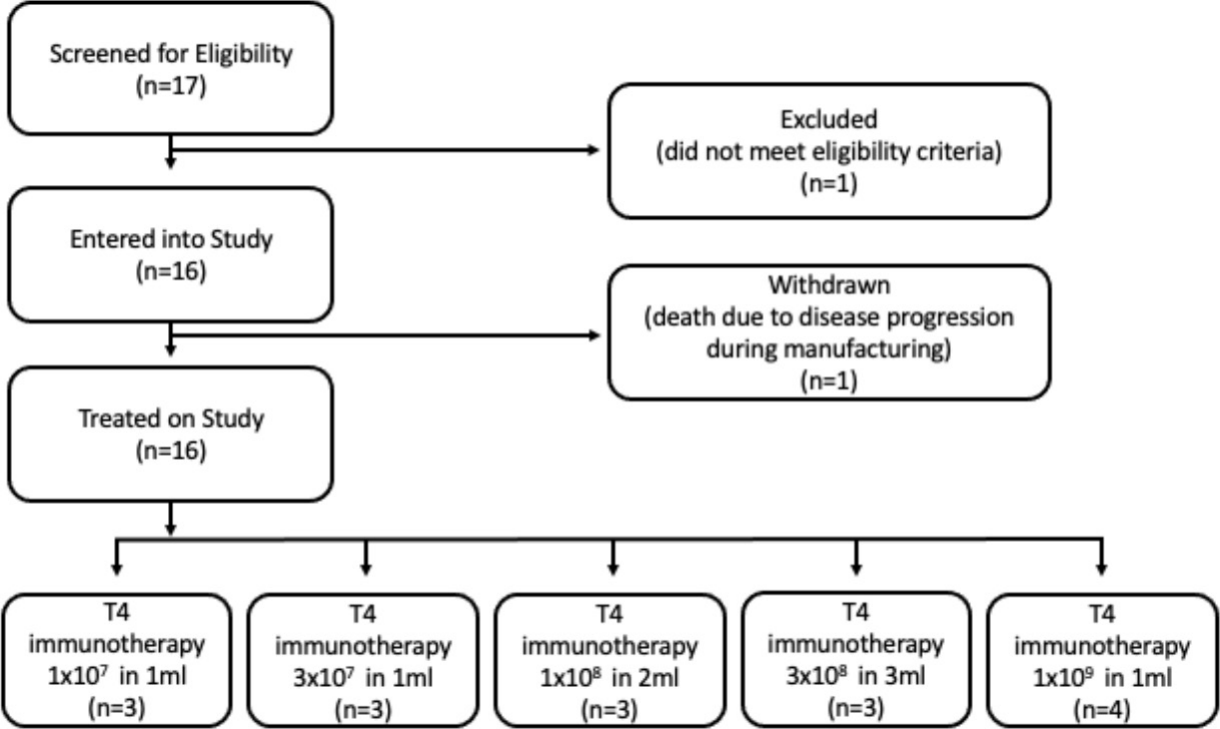
CONSORT (Consolidated Satndards of Reporting Trials) diagram.

Seventeen subjects consented to participate and were enrolled in the T4 immunotherapy phase 1 clinical trial between June 2015 and November 2018 (table 1). Fifteen subjects were treated across five dose cohorts ranging from 1×10^7^ to 1×10^9^ T4^+^ autologous cells. Two subjects did not receive T4 immunotherapy. One subject in cohort 2 died due to progressive HNSCC during the 14 day manufacturing process and a second subject in cohort 4 failed screening due to tumor proximity to major vessels (CONSORT diagram shown as figure 1). Ages of treated subjects was 39–82 years and ECOG Performance Status (ECOG-PS) ranged from 0 to 2. Subjects had received up to 8 lines of prior treatment with surgery, radiotherapy, and chemotherapy, in line with current clinical practice.

**Table 1:** Subject details

Cohort	Age (decade)	ECOG PS	Gender	T4 dose (×10^7^cells)	Primary site	Prior treatment
1	6th	1	M	1	Nasal cavity	Surgery×3; radical RT; CTX—Cisplatin, 5-FU; cetuximab
	6th	1	F	1	Oral (tongue)	Surgery×1; Radical RT; CTX—cisplatin, 5-FU
	6th	1	M	1	Oral (tongue)	Surgery×1; radical RT; CTX—cisplatin, 5-FU
2	6th	1	F	3	Oral (tongue)	Surgery×4; adjuvant RT; CTX—cisplatin, carboplatin; palliative RT
	6th	2	M	Died during T4 manufacture	Oral (tongue)	Radical RT; CTX—cisplatin
	6th	1	M	3	Oral (tongue)	Surgery×1; palliative RT; CTX×cisplatin, 5FU, cetuximab
	8th	2	M	3	Oropharynx	Radical RT
3	7th	1	M	10	Neck	Surgery×3; radical RT; CTX×cisplatin, 5FU, cetuximab, docetaxel, Carboplatin
	9th	0	M	10	Oral (buccal mucosa and mandible)	Surgery×4; Radical RT
	7th	1	M	10	Oropharynx	Surgery×1; radical RT; CTX×cisplatin, 5FU
4	7th	0	F	30	Oral (mandibular alveolus)	Radical RT
	4th	N/A	M	Failed screening	Oral (tongue)	Surgery×1; radical RT
	7th	N/A	M	30	Oral (oropharyngeal)	Surgery×1; RT×1 (nature unknown); CTX courses×1 (nature unknown)
	9th	1	M	30	Oral (tongue)	Surgery×1; RT×1 (dose unknown)
5	4th	1	M	100	Nasopharynx	Surgery×1; RT×1; CTX courses×1
	6th	1	F	100	Oral (mandibular alveolus)	Surgery×2; RT×2; CTX courses×1
	7th	1	M	100	Oropharynx	RT×1; CTX×3; pembrolizumab

CTX, chemotherapy; ECOG PS, Eastern Cooperative Oncology Group Performance Status; F, female; FU, follow-up; FU, follow-up; M, male; NA, not available; RT, radiotherapy.

### Manufacture of T4 immunotherapy

**Figure 2:**
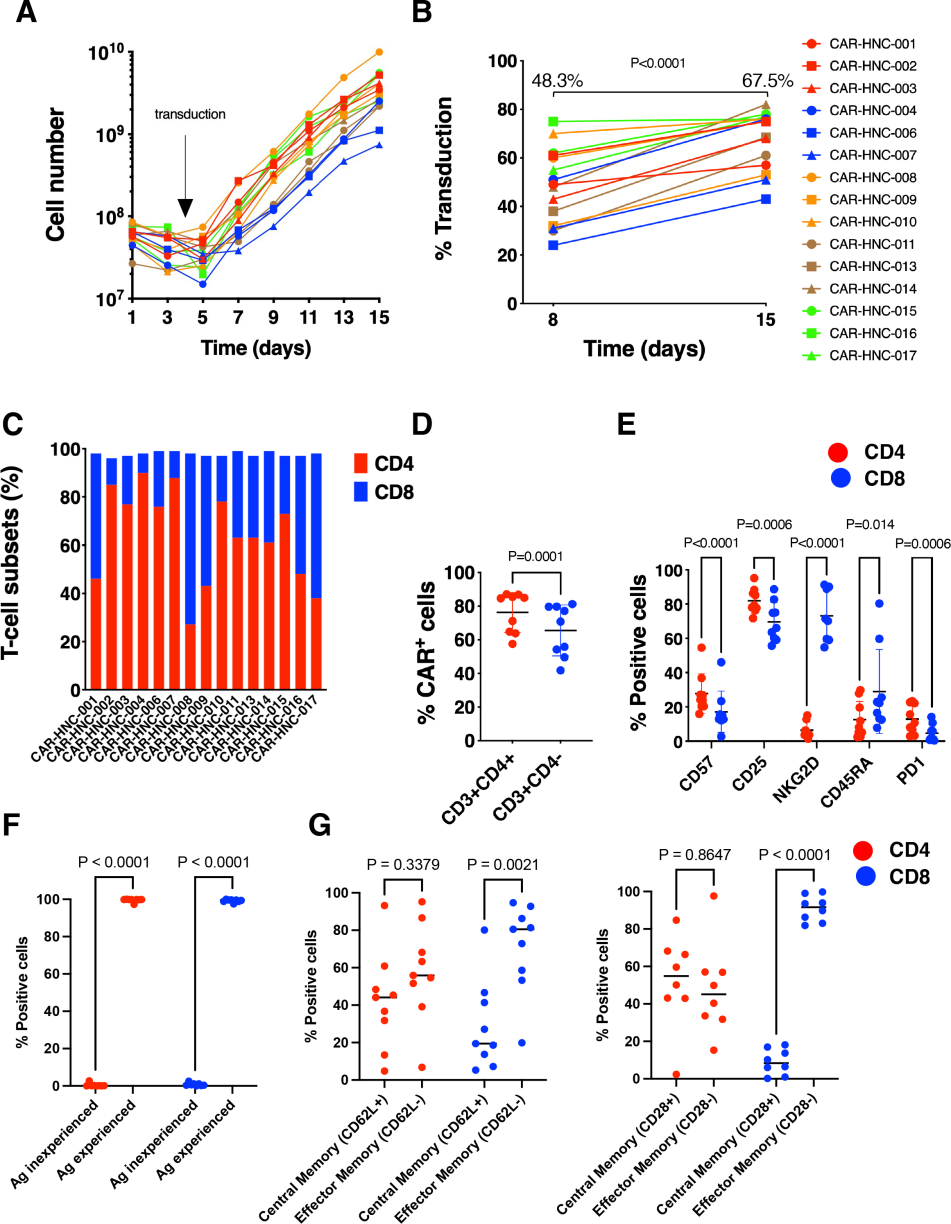
Manufacture of T4 immunotherapy. (A) Using blood (40–120 mL) as starting material the target cell dose was expanded successfully for all subjects without any batch failures. It should be noted that, in some cases, cells were discarded during manufacture to avoid waste of consumables, meaning that maximum yields were not obtained. (B) Proportion of transduced cells was analyzed by flow cytometry at the midpoint and end of the 2-week manufacturing process. Mean % T4^+^ cells are shown at each time point. (C) CD4/CD8 ratio of T4 CAR T-cell batches (n=9). (D) Transduction efficiency of CD4^+^ and CD8^+^ T-cells (n=9). (E–G) Expression of the indicated markers by T4 CAR T-cell batches (n=9). CD45RO was used to distinguish between antigen experienced (positive) and antigen inexperienced (negative) cells. All statistical analysis was by paired t-test.

T4 immunotherapy was successfully manufactured from whole blood (40–120 mL) in all cases, despite lymphopenia in 12 of 15-treated subjects (online supplemental table S4). There were no batch manufacturing failures. Robust expansion (figure 2A) and enrichment (figure 2B) of transduced cells in response to the bespoke 4αβ-cytokine receptor+IL-4 culture system was consistently observed,^8^ irrespective of starting material blood volume or lymphocyte count. Products were not formally tested for potency using functional assays (eg, cytokine release, cytotoxicity) prior to administration since cells were formulated as a fresh product.

Phenotyping of the T4 immunotherapy product indicated that CD4/CD8 T-cell ratio varied significantly across batches (figure 2C). Transduction efficiency was higher in the CD4^+^ subset (figure 2D), with significantly higher levels of expression of NKG2D and CD45RA in the CD8^+^ compartment (figure 2E). Individual batches showed antigen experience as illustrated by almost uniform CD45RO staining (figure 2F), with the CD4 CD45RO^+^ cells being evenly split between central and effector memory populations as assessed by CD62L or CD28 expression (positive or negative respectively; figure 2G). Analysis of the CD8 CD45RO^+^ cells using the same markers revealed a significantly higher population of effector memory cells (figure 2G).

### Systemic immune activation post-T4 immunotherapy injection

**Figure 3:**
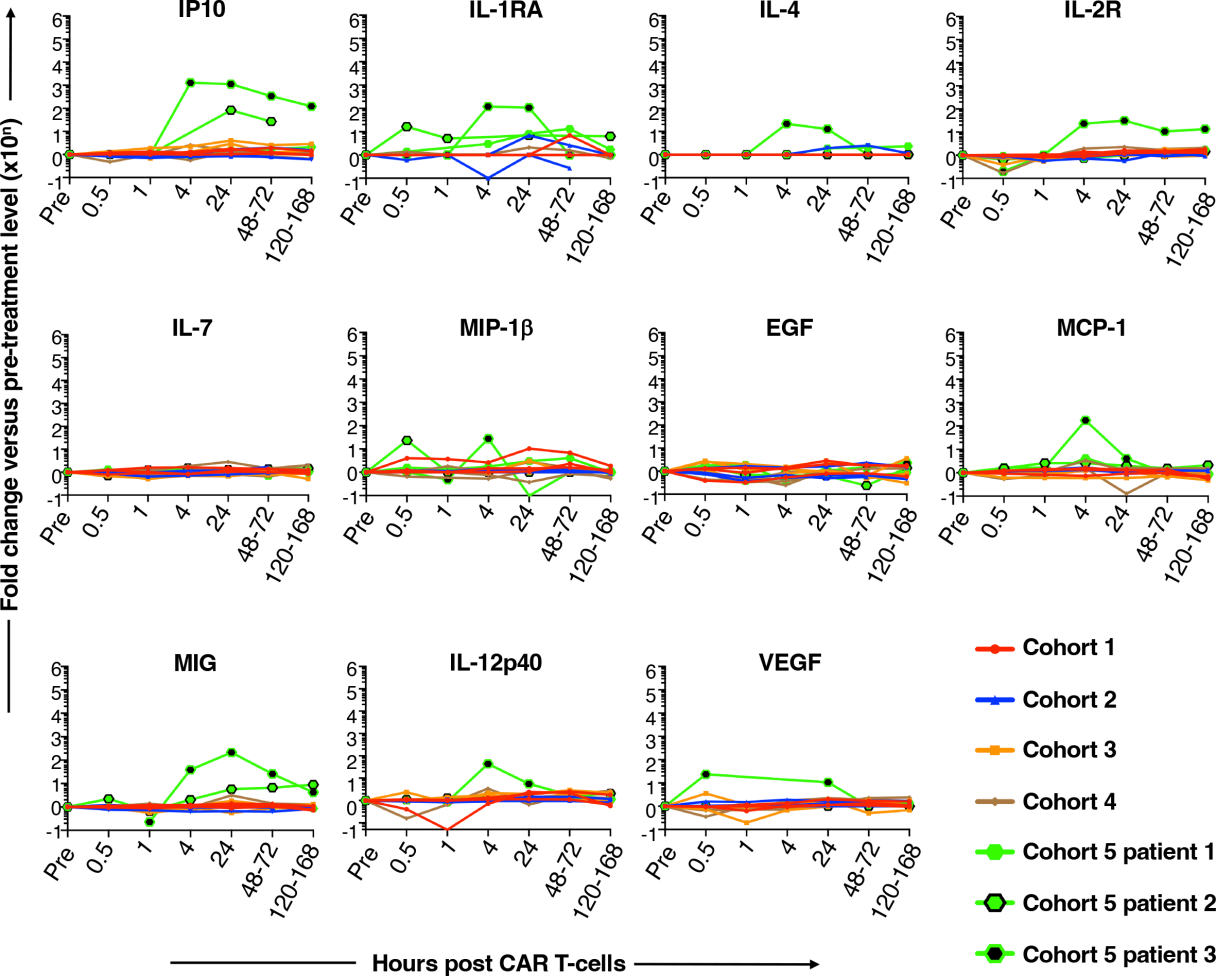
Circulating cytokine concentrations. Samples were collected pretreatment (pre) or at the indicated intervals post-CAR T-cell administration. Serial cytokine levels are shown in individual subjects, color coded for cohort number. Symbols are added to individual cohort 5 plots to enable distinction between these three subjects. The following cytokines were undetectable in all samples: fractalkine, granulocyte macrophage colony-stimulating factor, granzyme B, IFN-α, IFN-γ, IL-1α, IL-1β, IL-2, IL-5, IL-6, IL-8, IL-9, IL-10, IL-15, IL-17A, macrophage inflammatory protein 1α, regulated on activation, normal T cell expressed and secreted (RANTES) and TNF-α. CAR T-cell, chimeric antigen receptor T-cell.

Within hours of CAR T-cell immunotherapy treatment, elevated circulating cytokine levels were detected in two of the fifteen subjects treated, both of whom were in the highest (1×10^9^ cells) dose cohort (figure 3). Evidence of epitope spreading was interrogated utilizing MAGE-A3 and MAGE-A4 overlapping peptide stimulation of Peripheral Blood Mononuclear Cell (PBMC) pre-exposure and postexposure to T4 immunotherapy. Analysis was conducted for cohorts 3–5 (1×10^8^–1×10^9^ T4^+^ CAR T cells) with no definitive dose-dependent correlation observed (online supplemental figure 1A, B).

### Biopsy analysis

Paired pretreatment and post-treatment tumor core biopsies were obtained from subjects 8, 9, 10 and 17. Tumor was identified in only 2 of 4 post-treatment biopsies (subjects 8 and 10, obtained on days 8 and 15 after CAR T-cell treatment, respectively). In both cases, there was no evidence of EGF receptor downregulation in the post-treatment biopsy, nor were CAR T-cells identified using RNAScope (data not shown). IL-4 was not analyzed in tumor biopsies and was not used to select subjects for recruitment to this study.

### Clinical outcomes

**Figure 4:**
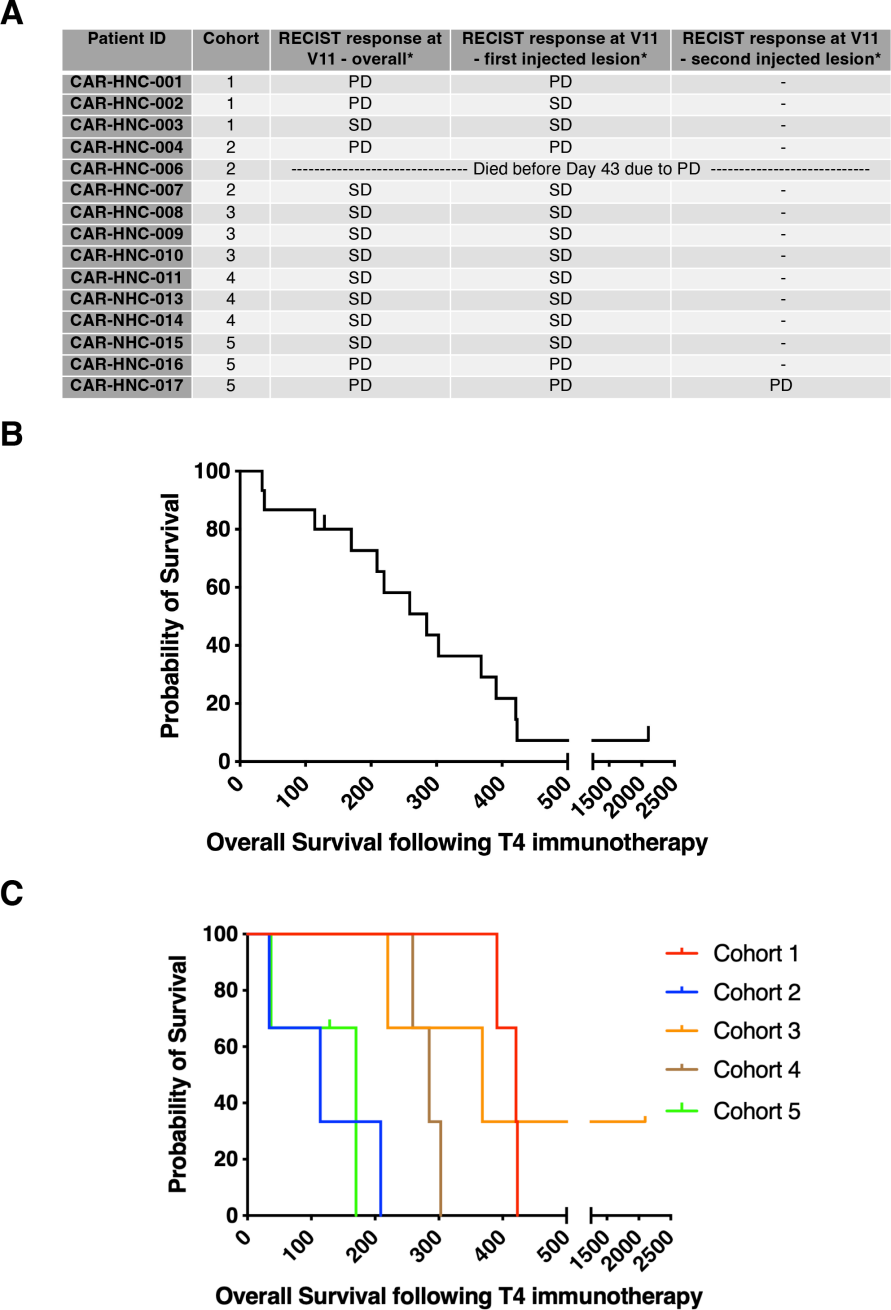
Patient outcome. (A) Outcome of patients according to cohort number.(B) Kaplan-Meier survival curve of all patients. Patient 6 died without receiving T4 immunotherapy and is not included. (C) Kaplan-Meier survival curve showing individual patient cohorts. PD, progressive disease; SD, stable disease.

No clinical responses by RECIST V.1.1 at day 43 were observed. Stable disease (SD) as best response was observed in 9/15 subjects (figure 4A) while progressive disease (PD) was observed in remaining subjects, giving a disease control rate of 60% at day 43. Median overall survival was 285 days (IQR 149–379 days), assessed with a data cut-off of December 9, 2022 (figure 4B). This compares favorably with historical expectations for refractory/untreatable HNSCC.^2^ There was no association between dose cohort and overall survival (figure 4C). One patient treated in cohort 3 achieved stable disease following T4 immunotherapy. He next received combination pembrolizumab and T-VEC treatment in a separate clinical trial and achieved a complete response, durable for over 3 years.

Spikes in neutrophil count and neutrophil/lymphocyte ratio (NLR; online supplemental figures 2A, B) were observed in the days immediately post T4 immunotherapy in the two highest dose cohorts. No such trend was seen with lymphocyte counts, which were low at baseline for the majority (online supplemental figure 2C).^16^ Spikes in C reactive protein and ferritin were also seen in a subset of subjects (online supplemental figure 2D, E), suggestive of an early systemic cytokine-driven response.

### Characterization of T-cell phenotype in stable versus progressive disease populations

**Figure 5:**
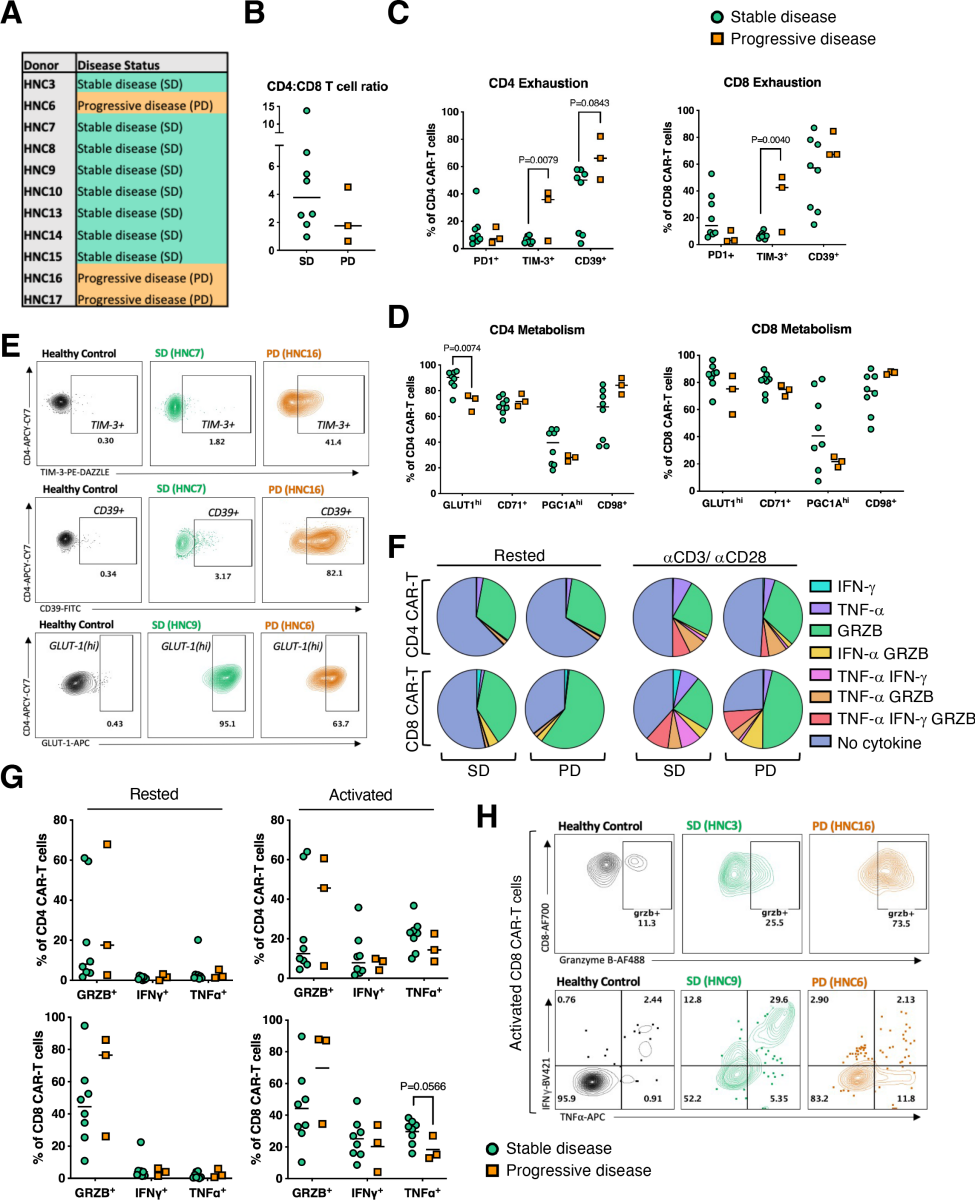
Immunophenotypic analysis of T4 CAR T-cell batches in patients with stable (SD) or progressive disease (PD). (A) Outcomes of 11 patients whose pre-infusion CAR-T phenotype was assessed in surplus retention samples. (B) Cryopreserved PBMCs from the infusion product were analyzed by flow cytometry, with gating on live CD4^+^ and CD8^+^ CAR-T cells. Ratio of CD4 to CD8 CAR-T cells in PD and SD patients is shown. (C) Frequency of PD1^+^, TIM-3^+^ and CD39^+^ CD4^+^ and CD8^+^ CAR-T cells. (D) Frequency of GLUT-1^hi^, CD71^+^, peroxisome proliferator-activated receptor-γ coactivator (PGC1A)^hi^ and CD98^+^ CD4^+^ and CD8^+^ CAR-T cells. (E) Representative flow cytometry gating of TIM-3^+^, CD39^+^ and GLUT-1^hi^ CD4^+^ CAR-T cells in SD and PD subjects with gating on healthy donor T-cells as a control. CAR-T effector molecule production was assessed by incubating cells overnight in the presence of brefeldin-A±plate-bound αCD3/αCD28 activation before staining for intracellular proteins. (F) Mean proportion of CD4^+^ and CD8^+^ CAR-T cells in SD and PD subjects that are single, double, or triple positive for Granzyme B (GRZB), IFN-γ and TNF-α; each pie chart totals 100%. (G) Total frequency of GRZB^+^, IFN-γ^+^ and TNF-α^+^ CD4^+^ and CD8^+^ CAR-T cells. (H) Representative flow cytometry gating of GRZB^+^, IFN-γ^+^ and TNF-α^+^ CD8^+^ CAR-T cells in an SD and PD subject with gating on healthy donor T cells as a control. Statistics: SD and PD groups were compared by unpaired t-test. CAR T-cell, chimeric antigen receptor; PBMCs, peripheral blood mononuclear cells.

In a subset of 11 subjects (3 PD and 8 SD; figure 5A) from whom sufficient CAR T-cells from the infusion product were retained, further immunophenotypic analysis was undertaken (gating strategy shown in online supplemental figure 3). The CD4/CD8 T-cell ratio in the T4 CAR-expressing population (eg, CD3^+^ CAR^+^) tended to be higher in the SD population (figure 5B). A more exhausted phenotype was observed at baseline in the three subjects with PD versus the eight who achieved SD (figure 5C). This was indicated by a trend toward higher CD39 expression by CD4^+^ CAR T-cells from the PD group, together with significantly elevated TIM-3 levels in both CD4^+^ and CD8^+^ CAR T-cells.

Next, markers of metabolite transportation and mitochondrial biogenesis in T4^+^ CAR T-cell products was examined. Significantly reduced GLUT1 expression was seen in the CD4^+^ CAR T cells of the PD population, suggestive of a reduced ability to uptake glucose for glycolysis (figure 5D). Differences in GLUT1 expression were not observed in the CD8^+^ CAR T-cells, while expression of CD71 (transferrin receptor) and CD98 (neutral amino acid transporter) were also similar in CAR T-cells from PD and SD groups. Peroxisome proliferator-activated receptor-γ coactivator (PGC)-1α promotes mitochondrial biogenesis and a trend toward increased expression of this factor was noted in CD4^+^ and CD8^+^ CAR T-cells from SD subjects (figure 5D). Representative dot plots of CD4^+^ CAR T-cells from a healthy control, a subject with SD and a subject with PD are shown for TIM-3, CD39 and GLUT1 (figure 5E).

Effector capacity of T4^+^ CAR T-cells was evaluated through analysis of GRZB, IFN-γ and TNF-α levels at rest and after overnight anti-CD3/CD28 stimulation. In SD subjects, we observed a trend toward an increase in CD8^+^ CAR T-cells that were double positive for these effector markers following overnight stimulation (figure 5F). In keeping with this, SD subjects also demonstrated a trend toward greater IFN-γ and TNF-α production by both CAR T-cell subsets after activation (figure 5G). Representative plots for CD8^+^ CAR T-cells that contain GRZB alone or co-produce IFN-γ and TNF-α are shown for a healthy donor, an SD and a PD subject (figure 5H).

While sample size is small, these data collectively suggest that more favorable clinical outcome is associated with a less exhausted and more functional CAR T-cell product.

### Safety of T4 immunotherapy

Overall, T4 Immunotherapy administered at doses ranging from 1×10^7^ to 1×10^9^ T4^+^ CAR T cells was safe and well tolerated. No DLTs or treatment-related adverse events (AEs) above grade 2 were observed (table 2). Most frequent treatment-related AEs included pain, swelling, fatigue and dysphagia. Two treatment-related serious AEs (SAEs) were reported in subjects treated at the highest (1×10^9^ T4^+^) dose level. Both consisted of a prolongation in protocol-mandated admission duration (24 hours) due to grade 1 fever. These were precautionary in both cases, in light of the possibility of evolving cytokine release syndrome (which did not ensue in either case). Three further SAEs unrelated to T4 immunotherapy were reported; namely oral pain, tumor hemorrhage and raised liver function tests (secondary to concomitant medication). All AEs and reactions are summarized in online supplemental table S5.

**Table 2:** Treatment-related adverse events (n=80)*

Toxicity	Grade 1, n (%)	Grade 2, n (%)	All, n (%)
Cardiovascular			
Tachycardia	1 (1.3)	0 (0)	1 (1.3)
Orthopnoea	1 (1.3)	0 (0)	1 (1.3)
Dermatologic			
Rash	1 (1.3)	0 (0)	1 (1.3)
Pruritus	1 (1.3)	0 (0)	1 (1.3)
Erythema	3 (3.8)	0 (0)	3 (3.8)
Gastrointestinal			
Nausea	1 (1.3)	0 (0)	1 (1.3)
Constipation	1 (1.3)	0 (0)	1 (1.3)
Diarrhea	1 (1.3)	0 (0)	1 (1.3)
Dysphagia	5 (6.3)	2 (2.5)	7 (8.8)
General			
Fever/fever	7 (8.8)	0 (0)	7 (8.8)
Swelling of injected tumor	10 (12.5)	4 (8)	14 (7.5)
Pain	9 (11.3)	8 (10)	17 (21.3)
Chills	2 (2.5)	1 (1.3)	3 (3.8)
Fatigue	6 (7.5)	5 (6.3)	11 (13.8)
Thick secretions	1 (1.3)	0 (0)	1 (1.3)
Hyponatraemia	1 (1.3)	0 (0)	1 (1.3)
Nasal discharge	1 (1.3)	0 (0)	1 (1.3)
Night sweats	1 (1.3)	0 (0)	1 (1.3)
Raised CRP	1 (1.3)	0 (0)	1 (1.3)
Prolonged admission	2 (2.5)	0 (0)	2 (2.5)
Hematologic			
Anemia	1 (1.3)	1 (1.3)	2 (2.5)
Infection			
Tumor	0 (0)	1 (1.3)	1 (1.3)
Bronchial	0 (0)	2 (2.5)	2 (2.5)
Neurologic			
Headache	1 (1.3)	0 (0)	1 (1.3)
Ptosis	1 (1.3)	0 (0)	1 (1.3)
Tinnitus	1 (1.3)	0 (0)	1 (1.3)

*No events above grade 2. Percentage shown is that of the 80 treatment-related adverse events.

CRP, C reactive protein.

Given the potential for on-target off-tumor activation of T4 through its engagement with ErbB family members in normal tissues, examination of leakage of T4^+^ cells into the circulation was undertaken. No signal above background was detected using quantitative PCR analysis of PBMCs from any patient at any time (data not shown). Moreover, using a bespoke flow cytometry assay conducted with fresh whole blood (online supplemental figure 4), no convincing peripheral leakage of CAR T-cells into the circulation was seen.

**Figure 6:**
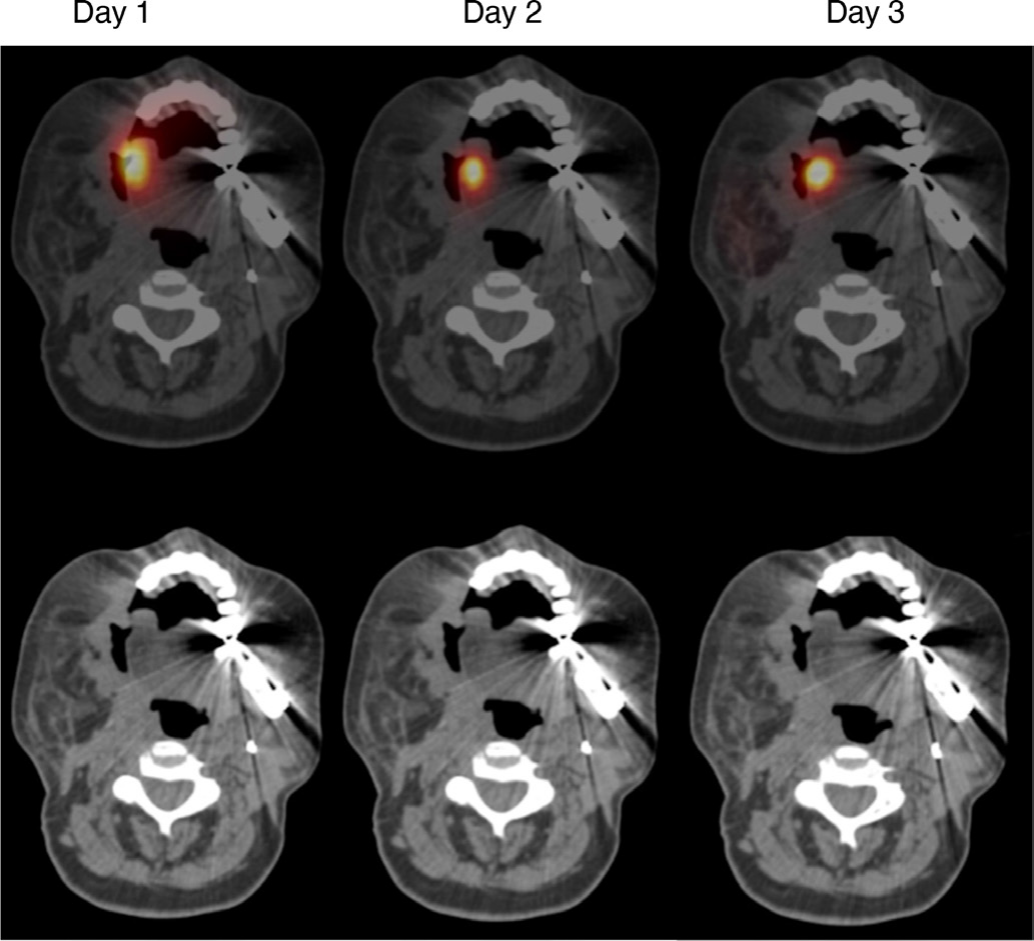
SPECT-CT imaging of [^111^In] radiolabeled T4 CAR T-cells. A radiotracer was prepared in which 30×10^6^ autologous T4^+^ CAR cells were labeled with 5MBq [^111^In] indium. The tracer was injected into a target lesion in the right buccal mucosa in a single subject (CAR-HNC-016). SPECT scans were performed at the indicated intervals and were coregistered with a CT scan (shown below) performed on day 1. CAR T-cells, chimeric antigen receptor T-cells; SPECT-CT, single photon emission-CT.

Intratumoral injection of an [^111^In] Indium passively labeled T4^+^ CAR-T radiotracer was undertaken on the same day as T4 immunotherapy administration in a single subject (CAR-HNC-016). The subject’s tumor was locally progressive in the right buccal mucosa. SPECT imaging was performed after approximately 1 hour and on days 2 and 3 postinjection. A CT scan was performed on day 1 and was coregistered with all three SPECT images. Imaging demonstrated focal retention of signal in the right buccal mucosa over 48 hours (figure 6). Whole body imaging showed no signal in other organs other than stomach (attributed to swallowed tracer, which had leaked from the site of injection).

## Discussion

This first in human dose escalation study was undertaken to investigate the safety and tolerability of a panErbB-targeted CAR T-cell product, T4 immunotherapy, in subjects with relapsed refractory HNSCC. We believe that this is the first published clinical trial of CAR T-cell immunotherapy for this indication. Despite previous issues with on-target off-tumor toxicity when ErbB family members have been targeted using CAR T-cell immunotherapy,^17^ safety of the intratumoral approach described here has been robustly demonstrated at doses of up to 1×10^9^ T4^+^ cells. The patient population was representative of relapsed refractory HNSCC with subjects over 80 years of age and with ECOG-PS up to two treated on study. The rapidly progressive nature of terminal stage HNSCC was exemplified by one disease-related death which occurred during the 2-week T4 manufacturing process.

No objective responses at 43 days were observed; 60% of subjects obtained disease control, with 9/15 having a best response of stable disease. There was no clear evidence of a dose–response effect, although target lesion size varied considerably, complicating the assessment of this relationship. Two patients had viable tumor in biopsies collected at days 8 and 15 post T4 immunotherapy. However, neither biopsy contained detectable CAR T-cells. This may reflect heterogeneity of CAR T-cell penetration into the tumors, sampling error or lack of persistence at these later time points. The rise in the NLR seen in the higher dose cohorts may reflect localized inflammation in the TME. It is possible that higher dose, and associated enhanced inflammatory responses, are detrimental to T4 immunotherapy efficacy. Further understanding of this as a potential mechanism of resistance could influence design of next generation edits to arm the CAR T-cells to better withstand the TME. However, the overall survival outcomes of the whole cohort were favorable when compared with historical controls, suggestive of an efficacy signal in this study.

Following intratumoral delivery of up to 1 billion CAR T-cells, leakage into the circulation was not detected. Moreover, a radiotracer prepared from the CAR T-cells remained at the site of injection for 48 hours. This is very likely to account for the lack of significant toxicity of intratumoral T4 immunotherapy. This also provides a strong rationale for further potentiation of this approach, for example, with cytokine armoring strategies^18^ since it is unlikely that such cells would gain access to the circulation in significant numbers. Lack of leakage of cells from the site of tumor injection into the general circulation, while favorable for off-tumor toxicity de-risking, may not be beneficial for clearance of metastatic or inaccessible disease.

Manufacture using a bespoke semiclosed fresh product platform was successful for all subjects from whole blood as starting material.^14^ Vein to treatment time was only 14 days owing to the delivery of a fresh product in which final sterility testing data was only available post-treatment. The CAR T-cell drug product demonstrated considerable donor to donor variability in immunophenotype, which is a recognized limitation of autologous CAR T-cell immunotherapy.^19 20^ We noted a tendency toward the generation of a CD4^+^ T-cell rich product from patients enrolled in this trial. The relative contribution of the manufacturing process and recruited patient population to this observation is uncertain. While the relative importance of each T-cell subset remains under debate, a number of studies have demonstrated the important role of CD4^+^ CAR T-cells in the control of both solid tumors and CD19-expressing malignancy.^21 22^ The analysis of phenotype and relationship with outcome was limited by numbers but is hypothesis generating that improved outcome may be linked to the administration of products with greater functionality and lower exhaustion marker expression.

Collectively these data demonstrate that T4 immunotherapy is safe as a single agent intratumoral therapy for HNSCC. Alone, it is insufficient to induce sustained tumor responses. This safety signal delivers a rationale for the addition of lymphodepleting chemotherapy prior to administration of T4 immunotherapy as the next step in derisking this approach. If the safety signal is maintained with systemic lymphodepletion, then further next generation edits to enhance T4 immunotherapy efficacy could be deployed. This next phase of the T4 immunotherapy trial has received regulatory approval and is currently ongoing.

## Data Availability

Data are available on reasonable request.
