# Supplementary material for: Intratumoral pan-ErbB targeted CAR-T for head and neck squamous cell carcinoma: interim analysis of the T4 immunotherapy study
Source: J Immunother Cancer. 2023 Jun 15;11(6):e007162. doi: 10.1136/jitc-2023-007162 (PMC10277526; doi:10.1136/jitc-2023-007162)
Supplement: Supplementary data [file jitc-2023-007162supp001.pdf]

Supplemental Materials

Intra-Tumoral pan-ErbB Targeted CAR-T for Head and Neck Squamous Cell Carcinoma: Interim Analysis of the T4 Immunotherapy Study

Papa et al

CONTENTS

1. **Supplemental Methods……………………………………………………………………2**

Flow cytometric monitoring of circulating T4^{+} CAR T-cells………………………….. 2

MAGE-A3/A4 Interferon-γ ELISPOT Assay……………………………………………2

Quantitative polymerase chain reaction (PCR) analysis of circulating T4^{+} T-cells...3

Analysis of core tumor biopsies…………………………………………………………4

2. **Supplemental Figures…………………………………….…………………………… 5**

Supplemental Figure S1 MAGE-A3 and MAGE-A4 ELISPOT analysis……….5

Supplemental Figure S2 Serial leukocyte counts, C-reactive protein, and ferritin post CAR T-cell immunotherapy……………………………………… 6

Supplemental Figure S3 Gating strategy used to prepare Figure 4…………...7

Supplemental Figure S4 Serial monitoring of circulating T4 cells by flow cytometry…………………………………………………………………… 8

3. **Supplemental Tables……………………………………………………………….... 9**

Supplemental Table S1. Release testing of T4 immunotherapy……………… 9
Supplemental Table S2. Dose and volume of T4 immunotherapy per cohort……………………………………………………………………………9
Supplemental Table S3. RNAScope probe for the SFG retroviral vector……..9
Supplemental Table S4. Circulating lymphocyte count prior to blood harvest………………………………………………………………………. 10
Supplemental Table S5. Summary of adverse events and reactions………… 10

4. Supplemental References…………………………………………………………….. 10

Supplemental Methods

Flow cytometric monitoring of circulating T4⁺ CAR T-cells

EDTA anticoagulated blood (50µL) was added to a FACS tube to which 4µL biotinylated anti-hEGF antibody (R&D systems, code BAF236) was added/ mixed for 15 minutes. Next, 1µL of Streptavidin-PE (ThermoFisher, code S866) was added/ mixed for 15 minutes. Then, 450µL of red blood cell lysis buffer (Biolegend 420301) was added, mixed and incubated for 15 minutes. Pre-mixed Countbright absolute counting beads (50µL; C36950, Invitrogen, Waltham, MA) were then added. Comparison was made to a positive control (T4$^+$ T-cells) and a negative control in which primary antibody had been omitted.

MAGE-A3/A4 Interferon-γ ELISPOT Assay

Analysis was performed using a human interferon (IFN)-γ T-cell Elispot assay (U-CyTech, Utrecht, The Netherlands). Thawed peripheral blood mononuclear cells (PBMC) were resuspended in RPMI (ThermoFisher Scientific)+10% AB serum (Sigma-Aldrich, Gillingham, UK). Triplicates of $1x10^6$ PBMC per well were co-cultured with peptide pools of MAGE-A3/A4 (Miltenyi Biotec, Bergisch Gladbach, Germany) at 2µg, 1µg and 0.5µg of each peptide/mL.

As positive controls, 1 or 5µL CEF (cytomegalovirus, Epstein Barr virus, influenza) viral peptide pool (Mabtech, Stockholm, Sweden) and 1 or 0.1µL of Infanrix -IPV+ Hib (GlaxoSmithKline UK Ltd, Brentford, UK) were used. Cultures (including unstimulated controls) were incubated overnight at 37°C. Cells were harvested, washed in RPMI +10% AB serum and transferred to an ELISPOT plate coated with the capture antibody. The plate was incubated at 37°C for 20-22 hours and cells were removed by washing as per manufacturer's instructions. Areas of cytokine capture were detected by addition of the biotinylated detection antibody. After washing, φ-labeled anti-biotin antibody (GABA) was added to each well and incubated for 1 hour at 37°C. Next, freshly prepared Activator I/II solution was added to each well and incubated at room temperature in the dark. Spot development was monitored every 5 minutes and the reaction was stopped once clear spots were visible by rinsing the wells with demineralized water. The plate was air dried at room temperature and spots counted using an immunospot analyzer (Bioreader®/EazyReader, Miami, FL). Mean values in test wells were compared with values in unstimulated control wells to calculate a stimulation index.

Quantitative polymerase chain reaction (PCR) analysis of circulating $T4^{+}$ T-cells

Peripheral blood samples were collected in EDTA tubes and genomic (g)DNA was extracted using the QIAamp DNA Mini Kit (Qiagen, Venlo, The Netherlands), according to manufacturer's instructions. A standard curve for transcript copy number was established by the amplification of linearized SFG T4, serially diluted from 10^{7} to 10^{2} copies of plasmid. The number of transgene copies per µg gDNA was determined using an Applied Biosystems 7500 Fast real-time PCR instrument (ThermoFisher) using labeled probes and primers, as described.[1]

Analysis of core tumor biopsies

Core tumor biopsies were obtained from 4 patients prior to and after CAR T-cell administration. Specimens were fixed in 10% (v/v) buffered formal saline and embedded in paraffin wax. Following review of the diagnostic hematoxylin and eosin section by a specialist head and neck pathologist for tissue adequacy, 4μM paraffin sections were routinely prepared. EGF receptor extracellular domain (Clone 3C6, Catalog No. 790-2988, Roche Tissue Diagnostics, Oro Valley AZ) and EGF receptor intracellular domain (Clone 5B7, 790-4347, Roche Tissue Diagnostics) immunohistochemical staining was undertaken using prediluted proprietary kit (Ventana Medical Systems, Roche Tissue Diagnostics) on a Ventana Benchmark Autostainer (Ventana Medical Systems, Roche Tissue Diagnostics) according to manufacturer's instructions. EGF receptor expression was quantified using the H-score method.[2] CAR T-cell presence was analyzed using mRNA in situ hybridization according to manufacturer instructions using a 2.5HD Assay-Brown (ACD, Bio-Techne, Abingdon, UK) and custom-designed probe against the SFG retroviral vector (Supplemental table 3). Positive and negative control probes used were Homo Sapiens Ubiquitin C and dapB (dihydrodipicolinate reductase gene of *Bacillus subtilis*; both ACD, Bio-Techne), respectively. Target retrieval time was optimized to 12 minutes and protease digestion to 30 minutes. Sections were counterstained with Hematoxylin QS (H-3404-100, Vector Laboratories, Newark, CA) and slides were mounted in VectaMount Permanent Mounting Medium (H-5000-60, Vector Laboratories).

Supplemental Figures

Supplemental Figure S1 MAGE-A3 and MAGE-A4 ELISPOT analysis. PBMC from the indicated subjects were isolated prior to and 29 days after T4 immunotherapy. Samples were stimulated with (A) MAGE-A3 and (B) MAGE-A4 peptide pools, making comparison with unstimulated control wells. Interferon-γ spots were enumerated and data expressed as a stimulation index with respect to unstimulated control wells.

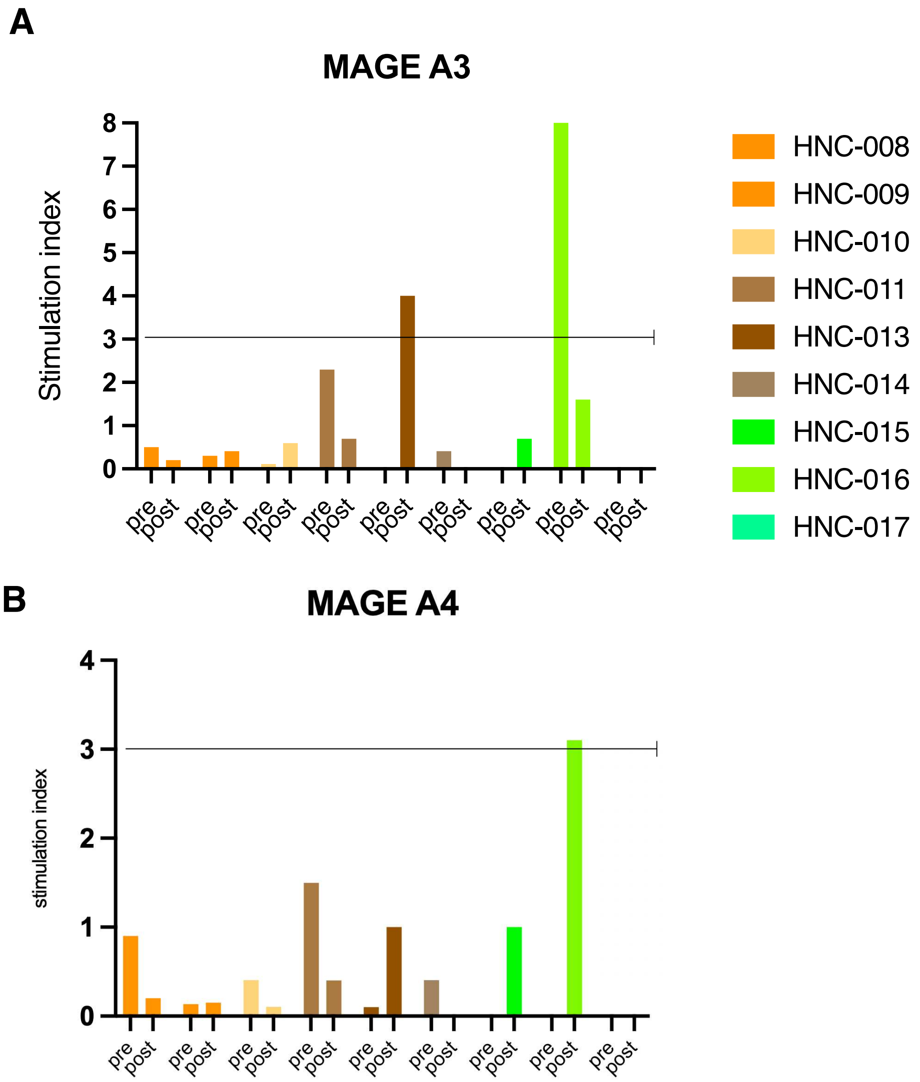

Supplemental Figure S2 Serial leukocyte counts, C-reactive protein, and ferritin post CAR T-cell immunotherapy. Neutrophil count (A), neutrophil to lymphocyte ratio (NLR; B), lymphocyte count (C), C-reactive protein (CRP; D) and ferritin (E) were measured in peripheral blood at the indicated timepoints following administration of T4 immunotherapy.

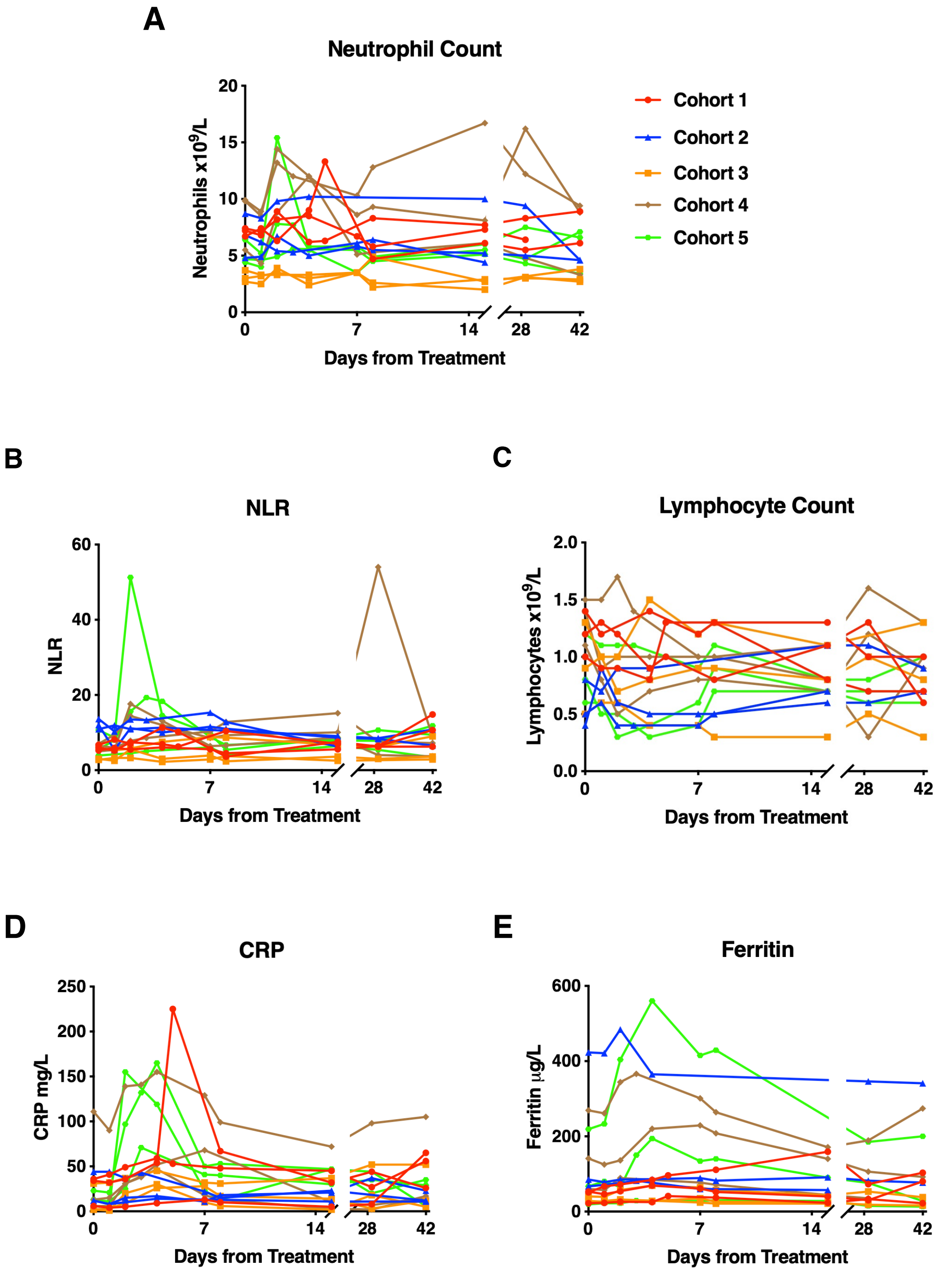

Supplemental Figure S3 Gating strategy used to prepare Figure 4. A healthy control and representative clinical grade batch (CAR-HNC16) are shown.

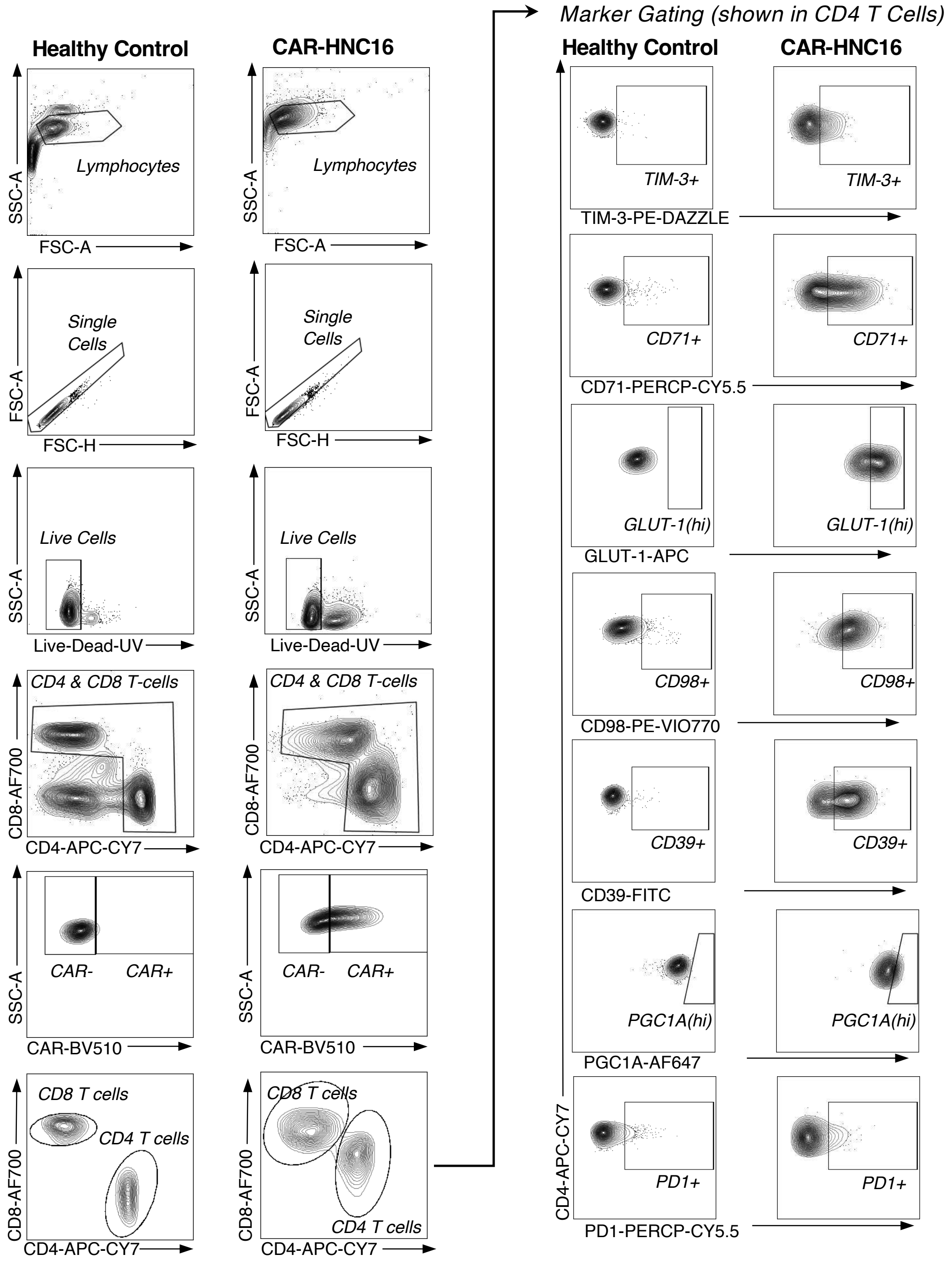

Supplemental Figure S4 Serial monitoring of circulating T4 cells by flow cytometry. Data from individual patients is shown. Absolute values were determined in whole blood using counting beads. Data are compared to values obtained when primary antibody was omitted (accounting for some negative values).

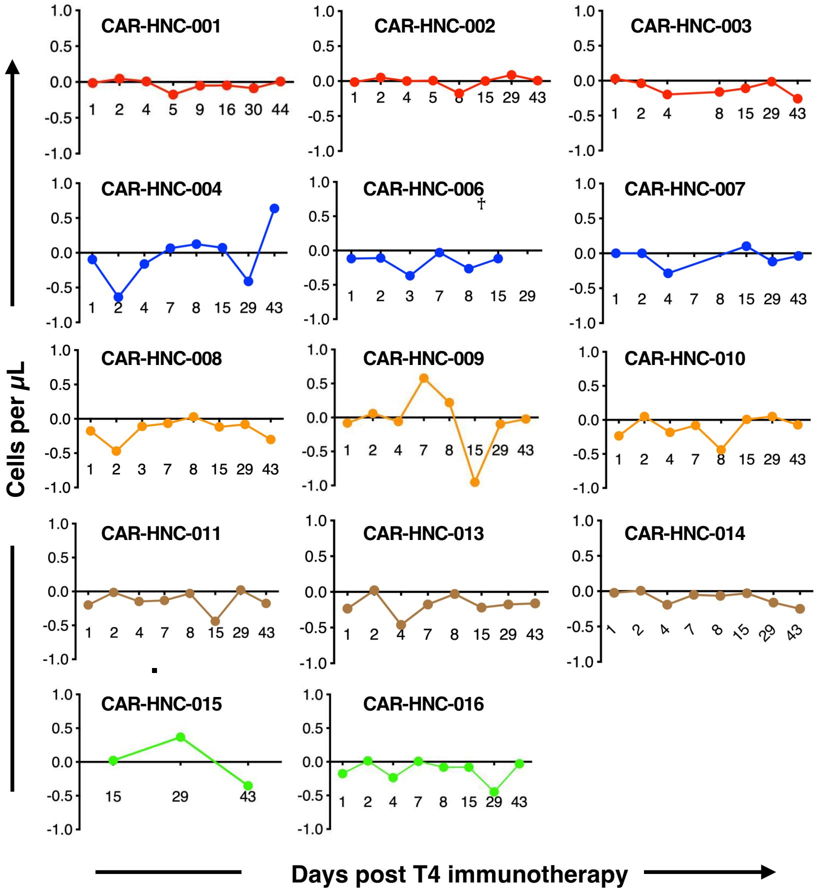

Supplemental Tables

Supplemental Table S1. Release testing of T4 immunotherapy.

Test	Test Method	Test Limits
Identity	Label	Correct identity of personal identifiers
	% T4$^+$ cells – stained with appropriate antiserum	Minimum 10% T4$^+$ transduced cells
Cell number	Flow cytometry using a Trucount tube	Minimum 1 x 10^7 cells. No maximum limit.
Viability	Flow cytometry (DAPI staining)	Minimum 70% viable cells within the lymphocyte gate
Sterility	BacT/ALERT & direct inoculation*	No growth
Mycoplasma Culture	Nucleic acid amplification technique*	Mycoplasma negative
Transgene function	Cell count on day 15 divided by cell count on day 3*	At least doubling in cell number achieved in response to culture in IL-4
Labeling efficiency**	Radiolabel incorporation	Minimum 30%

* Results of final sterility testing was not available at the time of product administration. All interim BacT/ALERT cultures and mycoplasma PCR tests had to be negative to permit product release.

** pertains to preparation of T4 radiotracer from an aliquot of the drug substance, which was undertaken in a single case.

Supplemental Table S2. Dose and volume of T4 immunotherapy per cohort.

Cohort	Target cell dose	Acceptable dose range of T4$^+$ cells	Volume for injection (mL)
-1*	3 x 10^6	3 x 10^6 cells	1 $\pm$ 0.2
1	1 x 10^7	3 x 10^6 - 10^7 cells	1 $\pm$ 0.2
2	3 x 10^7	1.1 – 3 x10^7 cells	1 $\pm$ 0.2
3	1 x 10^8	3.1 – 10 x10^7 cells	2 $\pm$ 0.4
4	3 x 10^8	1.1 – 3 x10^8 cells	3 $\pm$ 0.6
5	1 x 10^9**	3.1 – 10 x10^8 cells	4 $\pm$ 0.8

* -1 cohort if dose de-escalation in cohort 1 had been required

** Cell number was capped at 1 x 10^9 total cells

Supplemental Table S3. RNAScope probe for the SFG retroviral vector.

```
GATTAGTCCAATTTGTTAAAGACAGGATATCAGTGGTCCAGGCTCTAGTTTTGACTCAACAATATCACCAGCTGAAGCCT
ATAGAGTACGAGCCATAGATAAAATAAAAGATTTTATTTAGTCTCCAGAAAAAGGGGGGAATGAAAGACCCCACCTGTAG
GTTTGGCAAGCTAGCTTAAGTAACGCCATTTTGCAAGGCATGGAAAAATACATAACTGAGAATAGAGAAGTTCAGATCAA
GGTCAGGAACAGATGGAACAGCTGAATATGGGCCAAACAGGATATCTGTGGTAAGCAGTTCCTGCCCCGGCTCAGGGCCA
AGAACAGATGGAACAGCTGAATATGGGCCAAACAGGATATCTGTGGTAAGCAGTTCCTGCCCCGGCTCAGGGCCAAGAAC
AGATGGTCCCCAGATGCGGTCCAGCCCTCAGCAGTTTCTAGAGAACCATCAGATGTTTCCAGGGTGCCCCAAGGACCTGA
AATGACCCTGTGCCTTATTTGAACTAACCAATCAGTTCGCTTCTCGCTTCTGTTCGCGCGCTTCTGCTCCCCGAGCTCAA
TAAAA
```

Supplemental Table S4. Circulating lymphocyte count prior to blood harvest.

Cohort	Lymphocyte count ($x10^9/L$)*	T4 cell dose (x 10^6 cells)
1	0.9	10
	1.0	10
	1.3	10
2	0.5	30
	0.7	30
	0.6	30
	0.8	30
3	1.0	100
	0.6	100
	0.4	100
4	0.8	300
	N/A	300
	1.4	300
	1.2	300
5	0.7	1000
	0.8	1000
	0.9	1000

* Normal range 1.2 – 3.5 x 10^9/L

Supplemental Table S5. Summary of adverse events and reactions

	Number of events	Number of patients
Serious adverse events (SAE)	5	5
Of which:		
Suspected Unexpected Serious Adverse Reactions (SUSAR)	*0*	*0*
Other Serious Adverse Reactions (SAR)	*2*	*2*
Adverse events (not serious) (AE)	149	15
**CTCAE grade**:		
1	112	14
2	38	12
3	3	2
4	1	1
5	0	0

Supplemental References

1. Quintas-Cardama A, Yeh RK, Hollyman D, et al. Multifactorial optimization of gammaretroviral gene transfer into human T lymphocytes for clinical application. *Hum Gene Ther* 2007;18(12):1253-60. doi: 10.1089/hum.2007.088 [published Online First: 2007/12/07]
2. Mascaux C, Wynes MW, Kato Y, et al. EGFR protein expression in non-small cell lung cancer predicts response to an EGFR tyrosine kinase inhibitor--a novel antibody for immunohistochemistry or AQUA technology. *Clin Cancer Res* 2011;17(24):7796-807. doi: 10.1158/1078-0432.CCR-11-0209 [published Online First: 2011/10/14]
